# Docosahexaenoic Acid Reduced Vascular Endothelial Cell Injury in Diabetic Rats Via the Modulation of Autophagy

**DOI:** 10.34172/apb.2024.039

**Published:** 2024-03-20

**Authors:** Aysan Eslami Abriz, Atefeh Araghi, Mahdieh Nemati, Maryam Taghavi Narmi, Mahdi Ahmadi, Fatemeh Abedini, Rana Keyhanmanesh, Fariba Ghiasi, Reza Rahbarghazi

**Affiliations:** ^1^Drug Applied Research Center, Tabriz University of Medical Sciences, Tabriz, Iran.; ^2^Faculty of Veterinary Medicine, Amol University of Special Modern Technologies, Amol, Iran.; ^3^Stem Cell Research Center, Tabriz University of Medical Sciences, Tabriz, Iran.; ^4^Department of Physiology, Faculty of Medicine, Tabriz University of Medical Sciences, Tabriz, Iran.; ^5^Department of Applied Cell Sciences, Faculty of Advanced Medical Sciences, Tabriz University of Medical Sciences, Tabriz, Iran.

**Keywords:** Diabetes mellitus, Endothelial cells, Vascular system injuries, Docosahexaenoic acid, Autophagy, Rats

## Abstract

**Purpose:**

Among varied ω-3 polyunsaturated fatty acid types, the therapeutic properties of docosahexaenoic acid (DHA) have been indicated under diabetic conditions in different cell lineages. Here, we investigated the anti-diabetic properties of DHA in rats with type 2 diabetes mellitus (D2M) focusing on autophagy-controlling factors.

**Methods:**

D2M was induced in male Wistar rats using a single dose of streptozocin (STZ) and a high-fat diet for 8 weeks. On week 2, diabetic rats received DHA 950 mg/kg/d until the end of the study. After that, rats were euthanized, and aortic and cardiac tissue samples were stained with H&E staining for histological assessment. The expression of adhesion molecules, ICAM-1 and VCAM-1, was measured in heart samples using real-time PCR analysis. Using western blotting, protein levels of BCLN1, LC3, and P62 were measured in D2M rats pre- and post-DHA treatment.

**Results:**

Data showed intracellular lipid vacuoles inside the vascular cells, and cardiomyocytes, after induction of D2M and DHA reduced intracellular lipid droplets and *in situ* inflammatory response. DHA can diminish increased levels of ICAM-1 in diabetic conditions (*P*_Control vs. D2M rats_=0.005) and reach near-to-control values (*P*_Control vs. D2M rats_=0.28; *P*_D2M rats vs. D2M rats+DHA_=0.033). Based on western blotting, D2M slightly increased the BCLN1 and LC3-II/I ratio without affecting P62. DHA promoted the LC3II/I ratio (*P*=0.303) and reduced P62 (*P*_Control vs. D2M rats+DHA_ =0.0433; *P*_D2M vs. D2M rats+DHA_=0.096), leading to the completion of autophagy flux under diabetic conditions.

**Conclusion:**

DHA can reduce lipotoxicity of cardiovascular cells possibly via the activation of adaptive autophagy response in D2D rats.

## Introduction

 Atherosclerosis is a multifactorial degenerative inflammatory disease caused by the formation of plaques in the arterial wall under circumstances like hypercholesterolemia and type 2 diabetes mellitus (D2M).^1^ It was suggested that the interaction of low-density lipoprotein (LDL) with endothelial VCAM-1 led to the penetration of lipid compounds to the deeper layers of the vascular wall.^2^ The atherosclerotic plaques consist of vascular smooth muscle cells (VSMCs), inflammatory cells, lipids, and intracellular and extracellular debris.^1^ It has been indicated that the thinning of the fibrous cap causes the rupture of atherosclerotic plaques, and blood clotting, leading to clinical complications such as myocardial infarction, heart attack failure, stroke, and sudden death.^3^ To date, the management of atherosclerotic plaques includes the use of specific drugs (i.e. statins and cholesterol-regulating agents) and surgical approaches (angioplasty and bypass grafting).^4^

 A piece of research has pointed to the fact that autophagy plays a protective role in atherosclerotic conditions by regulating the activity of endothelial cells (ECs) and VSMCs.^5^ Several potential triggers for autophagy have been identified in atherosclerotic plaques, such as the accumulation of oxidized lipoproteins, inflammatory production of reactive oxygen species (ROS), and autophagic endoplasmic reticulum stress.^6^ Autophagy deficiency has been reported to accelerate the formation of atherosclerotic plaques in mice.^7^ The inhibition of the autophagy-related gene (ATG5) in macrophages can increase the possibility of atherosclerosis and apoptotic and necrotic changes in the arterial walls.^8^ Autophagy acts as a catabolic workhorse to clear lipids and dead cell debris via the formation of autophagosomes and further enzymatic digestion of lysosomes.^9,10^ These facts suggest that the induction of autophagy may be used as a potential strategy for the treatment of atherosclerosis.

**Figure 1:**
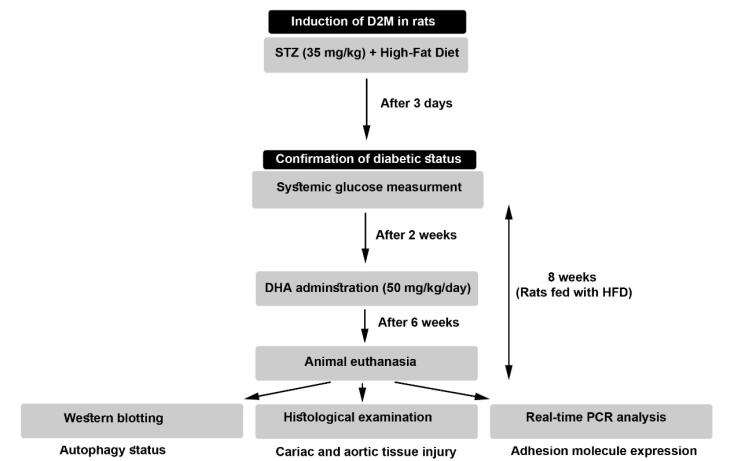
The flow chart of the experimental protocol

 It has been shown that docosahexaenoic acid (DHA), a natural omega-3 fatty acid, can exert therapeutic effects in several pathological conditions via the promotion of autophagic flux with simultaneous suppression of apoptotic changes.^11^ DHA is extracted from algal oil with anti-inflammatory compounds like atherosclerosis.^12^ This study was conducted to investigate the therapeutic effects of DHA on cardiovascular injury in D2M rats and its possible mechanism mediated via the autophagy signaling pathway (Figure 1).

## Material and Methods

###  Animal issues 

 To induce atherosclerotic injury, 60-day-old male Wistar rats (220 ± 20 g) were purchased from Med Zist Company (Tehran, Iran), and maintained under standard conditions (22 ± 2 °C, with a relative humidity of 50-60% and 12 hours light/12 hours dark cycles) for 7 days to acclimate the conditions. Animals were allowed to access tap water and chewing pellets. All experimental steps were approved by the local ethics committee of Tabriz University of Medical Sciences (IR.TBZMED.VCR.REC.1400.517). A total number of 24 rats were allocated into three groups (each in 8) including Control, D2M, and D2M + DHA groups.

###  Induction of D2M

 To this end, rats were fed with a high-fat diet (HFD) (48% carbohydrate, 20% protein, and 32% fat) in diabetic groups for 8 weeks, and control rats received a standard diet (67% carbohydrate, 22% protein, and 11% fat). To induce D2M-like conditions, HFD-fed rats received *i.p.* a single dose of streptozocin (STZ; 35 mg/kg; Sigma-Aldrich). Three to four days after injection of STZ, the glucose levels were determined in blood samples taken from the tail vein. Glucose levels of more than 300 mg/dL were considered diabetic. In both diabetic groups (D2M, and D2M + DHA) rats were fed with HFD until the completion of the experimental procedure. In the D2M + DHA group, rats received 50 mg/kg/d DHA (Cat no. D2534; Sigma-Aldrich) two weeks after induction of D2M until the completion of the experimental protocol (Figure 1). Finally, rats were euthanized using an overdose of ketamine and xylazine. Blood samples were directly taken from heart tissue and sera were isolated by centrifugation and stored at -80 °C until use. Heart samples and aortas were also taken for proteomic, histological, and gene expression analyses.

###  Monitoring autophagy status using western blotting 

 Protein levels of autophagy-related proteins such as BCLN1, LC3, and P62 were semi-quantitatively measured using western blotting. Cardiac tissue samples were lysed using RIPA buffer composed of 50 mM Tris-HCl (50 mM; Sigma-Aldrich), NaCl (150 mM; Merck), NP-40 (1% v/v), Sodium deoxycholate (0.5% w/v; Sigma-Aldrich), SDS (1% w/v; Sigma-Aldrich), EDTA (1 mM; Sigma-Aldrich) supplemented antiprotease cocktail (NaF 10 mM; Sigma-Aldrich). The samples were electrophoresed using 10% SDS-PAGE. After transfer onto the PVDF membrane (Millipore), the immunoreactive bands were detected using anti-BCLN1 (Cat no: sc-48341; Santa Cruz Biotechnology, Inc.), anti-LC3 (Cat no: 4775; Cell Signaling), and anti-P62 (Cat no: sc-10117; Santa Cruz Biotechnology, Inc.) antibodies. After phosphate-buffered saline (PBS) washes, the membranes were incubated with HRP-tagged secondary antibodies (Cat no: sc-516102; sc-2357 both purchased from Santa Cruz Biotechnology, Inc.). Using X-ray films and ECL solution (Bio-Rad), the immunoreactive bands were detected and normalized to housekeeping protein β-actin (Cat no: sc-48341; Santa Cruz Biotechnology, Inc.) using ImageJ (NIH; Ver. 1.4.). This assay was done in triplicate.

###  Histological examination

 After completion of the experimental protocol, heart samples were fixed in a 10% neutral buffered formalin solution (Merck) and paraffin-embedded. After deparaffinization and rehydration, 5-µm thick sections were prepared using a microtome (Leica). The procedure was followed by the staining of slides in hematoxylin and eosin (H & E) solution. The slides were visualized using an upright microscope (Model: ECLIPSE E100; Nikon) and imaged with (Model: UCMOS10000KPA).

###  Real-time PCR analysis of ICAM-1 and VCAM-1

 To see whether the administration of DHA can affect the expression of vascular adhesion molecules (ICAM-1 and VCAM-1) in diabetic rat cardiac tissue, real-time PCR analysis was performed (Table 1). In short, the samples were lysed in TRIzol reagent (Cat No: 0000124; MaxZol), and isolated RNAs were quantitated using the NanoDrop ND-2000 spectrophotometer, and reverse-transcribed into cDNA (Cat No: YT4500, Yekta Tajhiz Azma). PCR reactions were performed using a Roche Light Cycler 96 system and SYBR Green PCR kits (Cat No: YT2551, Yekta Tajhiz Azma). Relative transcription levels were calculated using the 2^−ΔΔCT^ formula after normalization housekeeping β-actin. Three sets of real-time PCR analyses were conducted.

**Table 1:** Set of primers used in this study

**Gene**	**Sequence**	**Tm (°C)**	**Ref**
ICAM-1	F: 5’-TGG AGG TGA CTG AGA AGT TGG-3’ R; 5’-CACAGTTACTTGGTCCCCTTC-3′	60	^13^
VCAM-1	F: 5- GTGTGTGAAGGAGTGAATCTGG-3′ R: 5’-CCAACAGCAGCACATGTCAGAA-3’	60	^13^
β-Actin	F: 5’-TCCCTGGAGAAGAGCTACG-3’ R: 5’- GTAGTTTCGTGGATGCCACA-3’	60	^14^

###  Statistical analysis 

 Data (Mean ± SD) of different groups were statistically analyzed and compared between the groups using One-Way ANOVA with Tukey post-hoc test. In this study, data were analyzed using GraphPad Prism ver. 8.4.3. *P* < 0.05 was considered statistically significant. All experiments were performed in triplicate otherwise stated.

## Results and Discussion

###  DHA promoted vascular autophagy response in D2M rats

**Figure 2:**
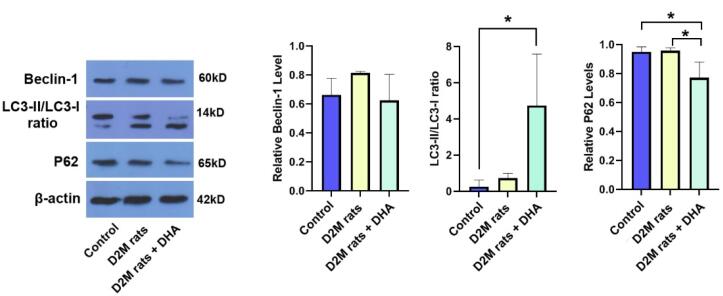
Monitoring autophagy response in diabetic rat cardiac tissue using western blotting that received DHA. Data indicated that the induction of D2M can non-significantly increase the BCLN1 and LC3-II/I ratio compared to the control group. Based on data, a 6-week administration of DHA increased autophagic flux by increasing the LC3-II/I ratio and reduction of intracellular P62 compared to the control and D2M rats. These data indicate that DHA can blunt the detrimental effects of D2M on autophagy response in rat cardiac tissue. (n = 3). One-way ANOVA with Tukey post hoc analysis. *P < 0.05

 It is thought that diabetic dyslipidemia can contribute to cardiovascular pathologies such as cardiomyocyte injury, atherosclerotic plaques, vascular cell calcification, and a reduction of vascular elasticity.^15,16^ *In situ* accumulation of monocyte-macrophage lineage in atherosclerotic lesions increases inflammatory response and lipid core, resulting in cardiovascular injury.^17^ Along with these statements, the management of atherosclerotic plaques remains critical in D2M-suffering patients. Here, the therapeutic effects of DHA were investigated on the autophagic response of cardiovascular cells in D2M male rats after 6 weeks. To see whether DHA can inhibit/stimulate the autophagy response in vascular cells under diabetic conditions, protein levels of BCLIN-1, LC3-II/LC3-I, and P62 were monitored 8 weeks after induction of D2M (Figure 2). Data indicated that despite the increase of BLCN1 in D2M rats however, these changes did not yield statistically significant differences when compared to the control group (*P* > 0.05; Figure 2). The administration of DHA in D2M rats exhibited relatively similar BLCN1 levels when compared to the control group. According to our data, D2M increased the LC3-II/-I ratio slightly without statistically significant differences as compared to the normal conditions (*P* > 0.05). Based on the data, we noted that the LC3-II/-I ratio significantly increased in D2M groups after the administration of DHA compared to control rats (*P* = 0.0303; Figure 2). Of note, these changes were not statistically significant between the D2M and D2M + DHA groups. Monitoring P62 levels indicated a lack of significant changes in P62 between control and D2M groups (*P* = 0.99), indicating that prolonged diabetic conditions in rats did not alter molecular machinery associated with lysosomal degradation and clearing injured cargoes. Data confirmed that DHA had the potential to significantly reduce intracellular P62 levels in the D2M group after administration of DHA, indicating the completion of autophagic flux (*P* = 0.0396; Figure 2). Administration of DHA in diabetic rats lowered the intracellular P62 contents compared to the control levels (*P* = 0.0433). These data demonstrate that the administration of DHA under diabetic conditions can stimulate adaptive autophagic flux associated with the increase of LC3-II/-I ratio and secretion of P62. The accumulation of excessive lipid droplets inside the cardiomyocytes and VSMCs indicates a defective autophagic response. In support of this notion, unchanged P62 levels show defective autophagy flux despite the initiation of molecular machinery related to autophagosome formation, leading to the inability of the cells to dispose of the lipid contents. Under diabetic conditions, overexpression of varied cytokines NF-κB, IL-1β, and TNF-α, along with an increase of ROS has been indicated.^18,19^ Based on previous data, it has been indicated that the oxidation of ATGs (ATG3, 7, and 10) and inactivation of PTEN by ROS under pathological conditions like D2M can suppress the autophagy response.^20^ In the present study, data confirmed that DHA stimulates autophagy by the induction of BCLN1 and LC3-II/I ratio and reduction of P62 in cardiac tissue of D2M-suffering rats. It is thought that P62 is a degradation substrate for autophagy, and its content is negatively correlated with the level of autophagy.^21^ It seems that the reduction of P62 and increase of LC3-II/I ratio is a possible accessible mechanism to eliminate excessive lipid content from cardiovascular cells during the D2M. In an experiment conducted by Shi and co-workers, they found that DHA can increase autophagic flux by the activation of LC3 and reduction of P62 levels in hypoxic mouse cardiomyocytes.^11^ It is well-established that DHA stimulates the autophagy through p53-mediated AMPK/mTOR signaling axis.^22^ The activation of AMPK in bone marrow macrophages promotes the deactivation of Toxoplasma gondii via the fusion of the autophagosome with parasite-containing vacuoles.^23^ These features indicate that microvascular cell lipotoxicity can be reduced during the D2M via the promotion of autophagy flux in a rat model.

###  DHA alleviated aortic lesions and cardiac tissue injury in diabetic rats

**Figure 3:**
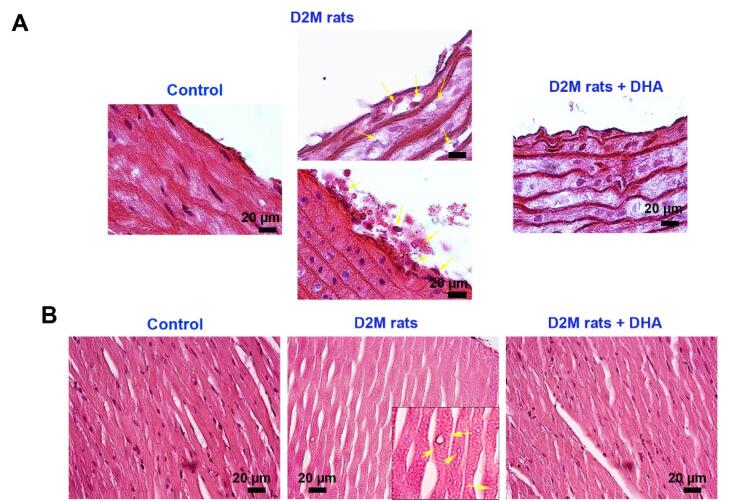
Histological examination of the aorta and cardiac samples in D2M after injection of DHA (**A-B**). Bright-field images show that the induction of D2M can lead to the accumulation of intracellular vacuoles (fat droplets; yellow arrows) in vascular smooth muscles, resulting in pathological hypertrophy (**A**). Along with changes, ECs at luminal surfaces are dislodged and detached from beneath the muscular layer after being exposed directly to diabetic serum (**A**). Data indicated that DHA can diminish EC injury and hypertrophic changes in tunica media after 6 weeks. The induction of D2M contributes to the accumulation of numerous small-sized intracardiomyocyte vacuoles. Data confirmed that DHA can reduce these changes and return to near-to-normal conditions

 Here, we performed H&E staining to monitor the histological changes of the cardiovascular system in rats after induction of D2M (Figure 3A). According to bright-field imaging, large-sized and delineated vacuoles containing fatty compounds were seen inside the VSMCs after feeding with the HFD regime (yellow arrows; Figure 3A). The progressive degenerative changes with deformed cell membranes were evident in ECs which are directly exposed to diabetic serum in which the integrity of these cells was missed leading to the recruitment of immune cells to the site of injury. Similar to the control rats, no remarkable changes can be detected in vascular cells belonging to DHA, indicating the lack of cytotoxicity for ECs (Figure 3A). Data points to the fact that the administration of DHA can reduce the intracellular accumulation of lipid droplets inside the VSMCs within the tunica media (Figure 3A). These features show that DHA can reduce the pathological changes induced by the D2M in the rat model via the regulation of intracellular lipid droplets. Monitoring the cardiac tissues revealed several intracellular vacuoles inside the cardiomyocytes of rats fed with HFD compared to the healthy control samples (yellow arrows; Figure 3B). It was suggested that the administration of DHA can reverse HFD effects and diabetic conditions in rat cardiomyocytes. These features demonstrate that DHA is a potent compound to blunt the detrimental effects of HDF + DM on rat cardiac tissue. It was previously suggested that DHA can diminish intracellular triglyceride inside hepatocytes via the down-regulation of cyclin D1 and increase of lipid β-oxidation.^24^ Consistently, Katsnelson and Ceddia found that treatment of rat skeletal muscle cells with 50 µM DHA can increase glucose and palmitate oxidation, leading to lipid and carbohydrate disposal from these cells, reduction of lipotoxicity, and carbohydrate damage.^25^ The increase in systemic glucose levels heightens the possibility of EC injury and dislodging orchestrated via progressive oxidative stress and inflammation.^26^

###  DHA reversed the up-regulation of vascular adhesion molecules in D2M rats 

**Figure 4:**
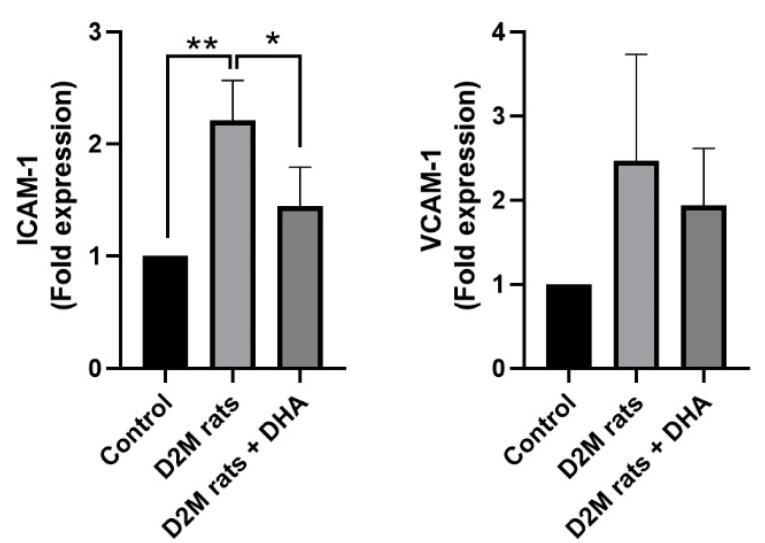
Monitoring the expression of ICAM-1 and VCAM-1 in diabetic heart samples using real-time PCR analysis. The induction of D2M increased the significant and non-significant expression of ICAM-1 and VCAM-1 in rat cardiac tissue compared to the healthy control rats. The application of DHA can diminish the unwanted expression of ICAM-1 and VCAM-1 and close to normal condition values. (n = 3). One-way ANOVA with Tukey post hoc analysis. *P < 0.05; and **P < 0.01

 Gene expression analysis revealed that the induction of D2M led to the up-regulation of ICAM-1 in rat cardiac tissues compared to the healthy control rats (*P* = 0.053; Figure 4). Despite the up-regulation of VCAM-1 in diabetic rats, these values did not reach significant levels compared to the control group (*P*> 0.05; Figure 4). We found that DHA administration in D2M rats reduced the expression of ICAM-1 and VCAM-1 and closed to near-to-normal levels. However, these changes yielded statistically significant differences in terms of ICAM-1 (*P* = 0.033) but not VCAM-1. These features indicate that the induction of D2M in a rat model is associated with the abnormal expression of vascular adhesion molecules such as ICAM-1 and DHA can reduce vascular abnormality by regulating these factors. It has been indicated that DHA has the potential to reduce intracellular levels of TNF-α, IL-6, and NF-κB levels in human ECs pre-treated to 1 mM palmitic acid in *in vitro* conditions.^27^ These data indicate the control of inflammatory response along with the regulation of lipid metabolism by DHA can reduce the detrimental effects of D2M in the cardiovascular system. Immune cells can attach to the inflamed diabetic endothelium layer and promote EC injury upon the up-regulation of adhesion molecules.^28^ Here, we found that DHA can reduce increased endothelial adhesion molecules, ICAM-1 and VCAM-1, in cardiac microvascular ECs. It is thought that the reduction of pro-inflammatory cytokines such as IL-1, IL-4, IL-6, and TNF-α by DHA reduces the expression of proatherogenic adhesion molecules.^29,30^ In response to DHA treatment, the release high mobility group box 1, and leukotriene B4 was shown to reduce in inflamed epithelial cells. Besides, Notch 1 and Jagged 1 factors were down-regulated in hypoxic and lipopolysaccharide-treated macrophages after being incubated with DHA.^31^ The inhibition of distinct signaling pathways especially Notch 1, can reduce M1 phenotype acquisition in monocyte-macrophage lineage.^32^ Therefore, reduction of inflammatory cytokines and prevention of active inflammatory phenotype are the possible therapeutic effects orchestrated by DHA in diabetic conditions. In contrast, DHA can promote the release of IL-10, and M2-type macrophage polarization with phagocytic activity.^33^ These effects are thought to reduce the close interaction of circulatory immune cells with the ECs while stimulate the elimination of excessive lipid content in the lumen of blood vessels.

## Conclusion

 The current study indicated that DHA can reduce atherosclerotic changes and relevant pathological in vascular ECs via the modulation of autophagic response. The reduction of vascular ECs can be associated with the reduction of adhesion molecules and activation of autophagic response. It is believed that the regulation of adhesion molecules can control the reciprocal interaction of immune cells with the endothelial layer while active adaptive autophagy behavior can eliminate the accumulation of excessive lipid content from the ECs and prevent the formation of atherosclerotic foci.

## Acknowledgments

 Authors wish to thank the personnel of the Faculty of Advanced Medical Sciences for their help and guidance.

## Competing Interests

 Authors declared that there is no conflict of interest related to this study.

## Ethical Approval

 All processes of this study were approved by the local ethics committee of Tabriz University of Medical Sciences (IR.TBZMED.VCR.REC.1400.517).
